# Molecular Basis of Yeasts Antimicrobial Activity—Developing Innovative Strategies for Biomedicine and Biocontrol

**DOI:** 10.3390/cimb46050285

**Published:** 2024-05-14

**Authors:** Ana-Maria Georgescu, Viorica Maria Corbu, Ortansa Csutak

**Affiliations:** 1 Department of Genetics, Faculty of Biology, University of Bucharest, Aleea Portocalelor 1-3, 060101 Bucharest, Romania; georgescu.ana-maria22@s.bio.unibuc.ro (A.-M.G.); viorica-maria.corbu@bio.unibuc.ro (V.M.C.); 2 Research Institute of University of Bucharest (ICUB), University of Bucharest, B.P. Hasdeu Street 7, 050568 Bucharest, Romania

**Keywords:** antimicrobial activity, killer toxins, carotenoids, red yeasts, biosurfactants, biocontrol, biomedicine

## Abstract

In the context of the growing concern regarding the appearance and spread of emerging pathogens with high resistance to chemically synthetized biocides, the development of new agents for crops and human protection has become an emergency. In this context, the yeasts present a huge potential as eco-friendly agents due to their widespread nature in various habitats and to their wide range of antagonistic mechanisms. The present review focuses on some of the major yeast antimicrobial mechanisms, their molecular basis and practical applications in biocontrol and biomedicine. The synthesis of killer toxins, encoded by dsRNA *virus-like* particles, dsDNA plasmids or chromosomal genes, is encountered in a wide range of yeast species from nature and industry and can affect the development of phytopathogenic fungi and other yeast strains, as well as human pathogenic bacteria. The group of the “red yeasts” is gaining more interest over the last years, not only as natural producers of carotenoids and rhodotorulic acid with active role in cell protection against the oxidative stress, but also due to their ability to inhibit the growth of pathogenic yeasts, fungi and bacteria using these compounds and the mechanism of competition for nutritive substrate. Finally, the biosurfactants produced by yeasts characterized by high stability, specificity and biodegrability have proven abilities to inhibit phytopathogenic fungi growth and mycelia formation and to act as efficient antibacterial and antibiofilm formation agents for biomedicine. In conclusion, the antimicrobial activity of yeasts represents a direction of research with numerous possibilities of bioeconomic valorization as innovative strategies to combat pathogenic microorganisms.

## 1. Introduction

**Figure 1:**
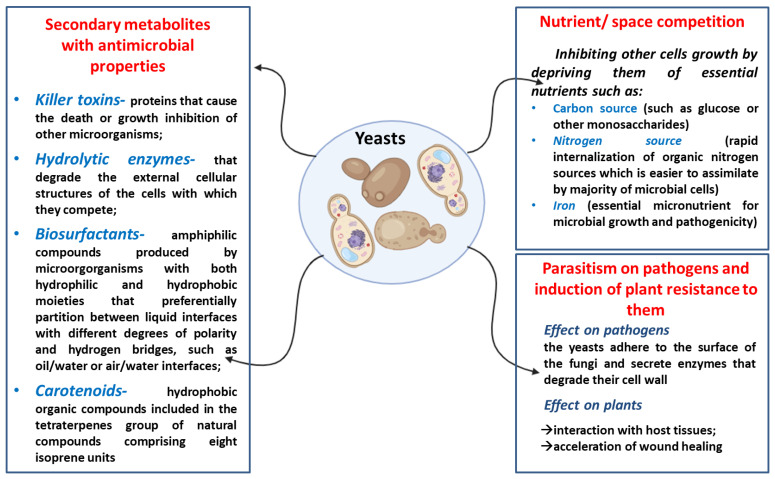
The main mechanisms of antimicrobial action described for yeasts.

Over the past decades, the development of new antimicrobial strategies aimed to combat the pathogenic microbial strains has become an intensely addressed research field. In the context of a significant abuse of synthetic chemical substances for limiting the contamination and the development of pathogenic microorganisms, the mechanisms of resistance to such compounds diversified, making the synthetic biocidal products almost inefficient [1]. The resistance of microbial pathogens to currently used biocidal substances is not relevant only for biomedicine but also for all associated activities which allow the propagation of resistant microorganisms almost out of control [2]. For example, in agriculture and animal husbandry, many chemically synthetized biocidal compounds are used in high quantities for crops and food protection against pathogens in order to meet global demand. In this circumstance, a new major threat to public health has arrived, namely the contamination of the food chain with antibiotics, which lead to the persistence of antibiotic-resistant pathogens which are further transferred to humans [2,3]. Therefore, discovering new and innovative alternatives has become not only an interesting research field but a necessity. Natural antimicrobial products, such as those derived from plants or microorganisms, represent a reliable opportunity to develop new antagonistic agents. Although known for some time, microbial derived antibacterial, antifungal and cytotoxic bioactivity agents remain an untapped resource which, if properly investigated, might represent the solution for this types of global concerns [4]. Therefore, a solution would be to investigate the relationships that are established in microbial communities in order to highlight the natural mechanisms by which they inhibit other microorganisms out of the simple desire to ensure their own survival. The yeasts, due to their widespread in most living environments, have developed a series of strategies for survival and occupation of the ecological niche. They possess numerous response mechanisms to stress conditions which allow them to compete for occupying the ecological niches [5]. Sometimes, this strategy is not sufficient, with some yeasts exhibiting various mechanisms of antagonistic action through which they determine the death of other microbial competitors or limit their growth. The most frequent antagonistic mechanisms of action are the competition for the substrate/nutrients from the environment, changing the composition of the culture medium or its physico-chemical characteristics and the synthesis of secondary metabolites that inhibit the development of other microorganisms (toxins, enzymes, volatile compounds, active compounds antioxidant) (Figure 1) [6,7]. Moreover, many yeast species are considered GRAS (Generally Regarded as Safe), showing no pathogenic characteristics under normal cultivation conditions. Moreover, they do not produce allergenic spores or toxins with a cytotoxic effect; they have a low degree of invasiveness and do not participate in the horizontal exchange of resistance genes. However, pathogenic yeasts might reach natural ecosystem via wastewater from hospitals that are not properly decontaminated or following direct transfer from infected man to environment. This is the main reason why yeasts strains from natural isolates sometimes present pathogenic properties [8]. Excluding the strains belonging to the genus *Candida*, the overall number of pathogenic yeasts is rather reduced, and the pathogenicity is preferably manifested in individuals with the system immune compromised [9,10]. The study of yeasts with antimicrobial activity is particularly important for different biotechnological branches such as ***agriculture***—for combating the phytopathogens, especially mycotoxin (aflatoxins, ochratoxins, deoxynivalenol, zearalenone, fumonisins a.s.o) producers since their impact on human health is severe due to their teratogenic, hepatotoxic, carcinogenic and immunosuppressive role [11,12]; ***food industry***—for combating (i) food pathogens such as *Pseudomonas aeruginosa*, *Aeromonas hydrophila*, *Salmonella enteriditis*, *Yersinia enterocolitica*, *Listeria innocua*, *Escherichia coli*, *Shigella flexneri*, *Salmonella typhimurium* [4] or *Bacillus cereus* [13], which cause gastrointestinal diseases after ingestion [4], or (ii) spoilage microorganisms which affect the quality of the food products; ***biomedicine***—for the development of new antifungal, antibacterial or anti-adhesive compounds mainly against pathogens that exhibit high resistance to commercially available antibiotic drugs; but also for ***fundamental research studies*** on the biology of eukaryotic cells, on their interactions or the interactions between eukaryotic and prokaryotic cells [4].

The present review deals with a detailed up-to-date insight in the potential use of the antimicrobial potential of yeasts for the development of new strategies aimed to counteract the growth of pathogens relevant for the biomedical field and for the agriculture. In terms of novelty, this review brings together important data regarding both the biosynthesis of antimicrobial compounds and their molecular mechanism of action against potential sensitive strains. In the first part of the review, the topic of killer yeasts is addressed, emphasizing the new yeast species in which this character was described and their biotechnological importance. For the second and the third part of the review, specific groups of yeasts are investigated. First, the subject of red yeast, an important group of yeast with huge economic potential, is discussed from the perspective of production of highly efficient antimicrobial agents. Then, the last part of the study addresses biosurfactant producing yeasts. Since the article of Santos et al. [14] named the biosurfactants as the biomolecules of the 21st century, a lot of attention has been directed towards them, proving their great versatility both as applications and as biological sources. Despite their huge biotechnological potential, the yeast biosurfactants are still not so well represented in the scientific research comparable with the one described for bacteria. The present review intends to fill in the gaps and to present distinct elements through which the antimicrobial potential of biosurfactants can be exploited.

Using two electronic databases (Google Scholar and PubMed) and specific keywords (“non-pathogenic yeasts”, “antimicrobial”, “molecular mechanisms”, “yeasts killer toxins”, “carotenoids”, “biosurfactants”), we identified more than 17,000 research papers published between 2010 and 2024. A rigorous selection process was implemented using inclusion and exclusion criteria based on specific yeast species derived from 300 scientific articles. Articles titles and abstracts were individually screened to select only the studies relevant to the topic, including intracellular or extracellular compounds produced by non-pathogenic yeasts with antimicrobial properties which can be furthered studied for biomedical and biocontrol applications. The specific inclusion criteria included validated taxonomical identification of yeasts: only research papers describing the antimicrobial activity of yeasts identified to the taxonomic level of species and/or genus were taken under consideration; standard methodology (derived from CLSI or EUCAST testing instructions) or highly reproducible methods used for describing the antimicrobial activity of the yeasts; access to the full-text article in English language using methods of obtaining in accordance with ethics and academic integrity.

## 2. Killer Yeasts

Killer toxins can be secreted by some yeast strains belonging to a variety of species, including *Saccharomyces cerevisiae*, *Kluyveromyces lactis* and *Whickerhamomyces anomalus* a.s.o. In general, killer toxins are able to determine death or inhibit growth of other microbial cells through a target-specific mode of action [15]. Chemically, the killer toxins are glycoproteins or extracellular proteins that can inhibit cell membrane functions in susceptible pathogenic yeasts or other microorganisms, a process mediated by specific receptors present in the cell wall of susceptible microorganisms [16]. From a genetic point of view, the killer toxins can be encoded by dsRNA molecules, DNA plasmids or by chromosomal genes.

### 2.1. Yeast Species with Killer Potential Determined by dsRNA Molecules

**Figure 2:**
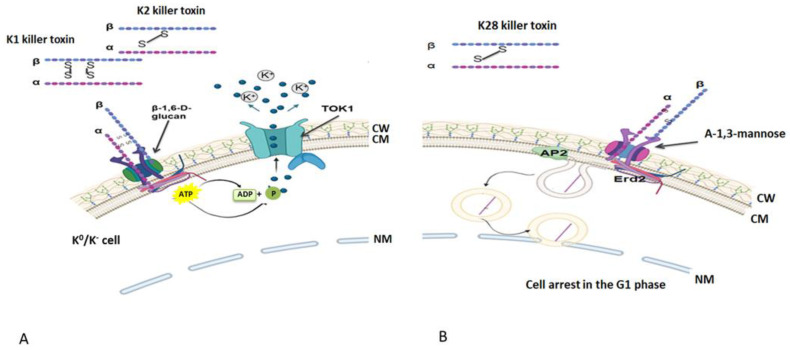
The mechanism of action of the (**A**) K1, K2 and (**B**) K28 killer toxins described for *S. cerevisiae* (figure created using Biorender.com, accessed on 30 March 2024) (CW—cell wall; CM—cell membrane; NM—nuclear membrane).

*S. cerevisiae* is, probably, the best-known yeast species with killer potential. Its killer activity has been described for the first time at the beginning of the 1960s when it was proven the existence of some intracytoplasmatic dsRNA molecule responsible with the encoding of the killer toxins K1, K2 and K28 and the appropriate immunity factor [17,18] (Figure 2). Mature K1, K2 and K28 toxins are bridge-stabilized α/β heterodimers disulfides that recognize certain binding sites present in the cell wall of cells of sensitive yeast. The toxins K1 and K2 recognize and bind without consumption of ATP to β-1, 6 D-glucan receptor, while K28 specifically recognizes α-1,3-mannanoproteins. The translocation of the toxin to the plasma membrane is an ATP-consuming process and involves interaction with a secondary receptor (for the K1 and K2 toxins, the α subunit interacts with a membrane receptor named Kre1p, while in the case of K28, the toxin interacts with Erd2). Both K1 and K2 toxins cause death of the susceptible cell by increasing the membrane permeability to H^+^ and the uncontrolled release of K^+^ ions and other molecules such as amino acids or glucose (by activating the TOK1 pumps, which are potassium channels located in the yeasts plasma membrane involved in the maintenance of the membrane potential under chemical stress) [19,20]. After binding to the specific receptor, the K28 toxin is internalized by endocytosis, a process mediated by the AP2 protein, and reaches the nucleus of the sensitive cell where it disrupts the process of division, causing cell arrest in the G1 phase and, subsequently, the death of the sensitive yeast cell [21]. After *S. cerevisiae*, killer toxins encoded by dsRNA have been described for several other yeast species, including *Hanseniaspora uvarum*; *Zygosaccharomyces bailii* and *Ustilago maydis*.

***H. uvarum*** belongs to a group of species important in the process of alcoholic fermentation of fruit, especially grapes. Strains of *H. uvarum* have also been identified in cashew nut juice, palm wine, tequila, cider, sugar cane spirits, coffee and cocoa. However, in other food products such as honey, yoghurt, orange juice and beer, it is considered as a spoilage species [22]. The extracellular protein toxin secreted by killer strains is considered deadly to susceptible yeast strains, which usually belong to other yeast species, filamentous fungi or even bacteria [23]. In a study by Radler et al. [24], it was found that some strains of *H. uvarum* produced the killer toxin KT470 as well as the toxin K1, highly similar to the K1 toxin described for *Saccharomyces* species. Although the secreted killer toxins (KT470 and K1) have an identical molecular weight (18 kDa each), the dsRNA encoding the corresponding toxins differs in size [25]. However, the relatively small (1.0 kb) dsRNA from *H. uvarum* encodes the entire 18 kd killer toxin KT470, which probably consists of less than 200 amino acids [22]. The *H. uvarum* killer toxin has applications in biomedicine and biocontrol. Hameed, A. [23] determined whether or not this species would inhibit growth of different bacterial and yeasts strains belonging to the *Staphylococcus aureus*, *Klebsiella pneumoniae*, *E. coli*, *P. aeruginosa*, *Rhodotorula mucilaginosa* and *Candida tropicalis* species. According to the results presented, the highest zone of inhibition and the highest potential to produce killer toxins by *H. uvarum* was observed at pH 4. At pH higher than 4.5, the killer toxin loses its activity.

***Z. bailii*** have the ability to spoil many foods and beverages, especially those with low pH and high sugar content. The characteristics that contribute to this virulence are assimilated with the product composition and processing methods [26]. Killer toxin-secreting strains of the yeast *Z. bailii* have been shown to contain linear double-stranded RNA (dsRNA) that persists in the cytoplasm of the infected host cell as encapsulated virus-*like* particles. L and M dsRNAs have been associated with the major capsid protein of 85 kDa, while the additional dsRNA (2.8 kb) is present only in the *Z. bailii* killer strains and is encapsulated by a coat protein of 35 kDa size [27]. The toxin named zygocin is produced and secreted by a strain infected with the osmotolerant yeast killer virus. The toxin can inhibit the growth of phytopathogenic filamentous yeasts and fungi. The viral toxin interacts with the receptors from the cell wall and plasma membrane. Zygocin receptors have been isolated and partially purified from the mannoprotein fraction of the yeast cell wall [28]. They were successfully used, by receptor-mediated affinity chromatography, as a biospecific ligand for the efficient purification of the 10 kDa protein toxin in a single-step study conducted by Weiler, F. [28]. However, the study by Radler et al. [24] showed that the killer toxin produced, named KT412, was highly stable at pH 3 at 4 °C. According to Weiler et al. [28] and Radler et al. [29], *Z. bailii* has been shown to have a strong inhibitory effect on several species of both yeasts and filamentous fungi, such as *Candida albicans*, *C. krusei*, *C. glabrata*, *Sporothrix schenckii*, *Fusarium oxysporum*, *S. cerevisiae*, *K. marxianus*, *H. uvarum*, *W. anomalus*, etc.

***U. maydis*** is a filamentous fungus considered phytopathogenic, mainly affecting maize leaves, causing tumors [30,31,32]. There are three lethal toxins harbored by *U. maydis* and three toxin resistance genes. Each killer strain affects the susceptible strains and the other two killer strains but is resistant to its own killer toxin. The KP4 and KP6 toxins were well characterized, and the crystal structure of the KP4 toxin was determined. The M2 dsRNA segments of each subtype encode KP4 and KP6 toxins. The KP4 toxin is a monomer of 11.1 kDa, while the KP6 toxin comprises two polypeptides: CI (8.6 kDa) and p (9.1 kDa) [33,34]. The KP6 toxin is expressed as a pre-protoxin and is converted to a protein by the protease Kex2p. However, expression of KP4 toxin does not require Kex2p activity. KP4 and KP6 toxins were expressed from cDNA clones. One of the three toxins, namely KP1, is the least fully understood because of its low level of expression [30]. The structure of KP4 was the first established followed by that of KP6. The KP4 toxin is a single polypeptide with 105 amino acids and is not processed by Kex2p (unlike KP1 and KP6). There is no sequence similarity between KP4, KP6 and other known killer toxins. The KP4 toxin is highly basic, with a pH above 9.0, whereas most yeast toxins are acidic. On the other hand, the KP6 and KP1 toxins have a neutral pH [35].

### 2.2. Yeast Species with Killer Character Determined by dsDNA Plasmids

**Figure 3:**
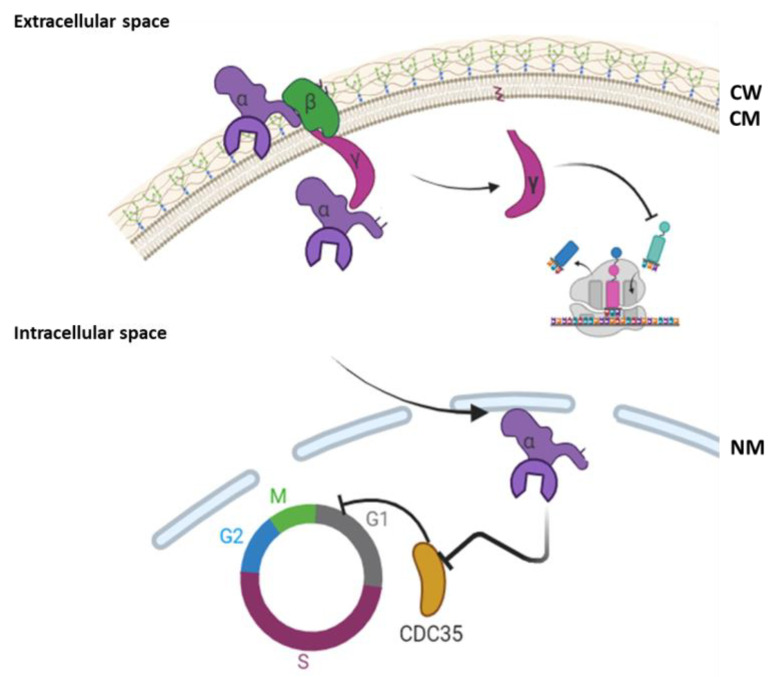
Mechanism of action of zymocine produced by *K. lactis* strains (figure created using Biorender.com, accessed on 30 March 2024) (CW—cell wall; CM—cell membrane; NM—nuclear membrane).

Other yeast species produce killer toxins encoded by dsDNA plasmids. The best-known species from this point of view is *K. lactis*. In general, members of the genus *Kluyveromyces* belong to the class Ascomycetes [36]. The synthesis of the killer toxin in *K. lact* is involves the existence of two intracytoplasmic dsDNA plasmids named pGKL.1 (8.9 kpb) and pGKL.2 (13.4 kpb). pGKL.1 dsDNA encodes the killer toxin, the immunity factor and a DNA polymerase. pGKL.2 dsDNA contains mainly genes involved in its own replication, respectively, the replication of pGKL.2 plasmid and in the transcription and pre-mRNA processing [37]. The killer toxin produced by *K. lactis* is named zymocine and has a trimeric structure formed by three subunits: the α-subunit, which presents a chitin binding domain and an enzymatic domain with chitinase function; the β-subunit, a highly hydrophobic domain; and the γ-subunit, which presents cytotoxic effect. The zymocin mechanism of action is different depending on the species attacked, but at present, the entire mechanism of action is not yet fully understood. When zymocine interacts with a *S. cerevisiae* cell, the toxin is attached to the cell wall through the chitin binding domain of the α-subunit. Then, the β-subunit facilitates the internalization of the α and γ subunits due to its high affinity for the cell membrane. After internalization, the trimer is destabilized by breaking the disulfide bonds, the γ-subunit degrades tRNA, thus blocking the translation process, while the α-subunit enters inside the nucleus where it determines the repression of the *CDC35* gene transcription, causing cellular arrest in the G1 phase of the cell cycle. When the zymocine interacts with a *Candida* cell, besides the previously described mechanism, the chitinase domain of the α-subunit directly interacts with the cell wall, causing its destabilization [38,39,40] (Figure 3).

***K. wickerhamii*** is a species basically isolated from various sources, such as spontaneously fermented dairy products from tropical areas and from grape must [41]. The killer toxin identified for this species is Kwkt, with a size of 72 kDa. It is a toxin that seems to have a much more pronounced inhibitory effect on species belonging to the *Brettanomyces/Dekkera* genera. It is also worth mentioning that the mechanism of action of this toxin is still not very well understood.

***K. siamensis*:** Members of this species are mainly isolated from mangrove roots and are able to produce a killer toxin of 66.4 kDa, highly active against the pathogenic yeast *Metschnikowia bicuspidate*, which affects crab populations [42]. In the same study, conducted by Buzdar et al. [42], it was found that the strain *K. siamensis* produced the highest amount of killer toxin at pH 4 in a concentration of 0.25% NaCl, incubated at 25 °C.

***Wingea robertsiae (syn: Debaryomyces robertsiae)*** has two cryptic linear plasmids: pWR1A (8.3 kb) and pWR1B (14.6 kb). In the study of Klassen and Meinheardt [43], two *W. robersiae* strains, one carrying the plasmid and one without the plasmid, were prepared to study the killing effects of concentrated culture supernatants in liquid media [43]. Furthermore, chitin affinity chromatography and Western blot analysis showed the expression and secretion of a specific plasmid protein resembling the subunit of the *K. lactis* killer toxin. Most likely, the mechanism of action of the identified killer toxins (pWR1A and pWR1B) is that of cell arrest in the G1 phase of the cell cycle [43]. The antibacterial activity of this strain has not been associated with stress conditions, i.e., pH variations, high salt concentrations, temperature variations, etc., and has so far only been shown to be effective on *S. cerevisiae* strains.

### 2.3. Yeast Species with Killer Toxins Encoded by Chromosomal Genes

The main yeast species known for its killer toxin encoded by chromosomal genes is *W. anomalus* (syn. *Pichia anomala*). Members of this species produce several types of killer toxins with molecular weights between 8 and 300 kDa. These toxins are encoded by chromosomal genes, fact proved during experiments in which the presence of acridin orange or cycloheximide did not affect the production of killer toxin. The killer toxins described for the members of this species differ through the spectrum of activity and physico-chemical properties (Table 1).

***S. cerevisiae:*** Chromosomal encoded killer toxins secreted by *S. cerevisiae* strains are (i) KHR (Killer of Heat Resistance)—gene on chromosome IX, and (ii) KHS (Killer of Heat Susceptibility)—gene on chromosome V, CF8 and M12. The KHR toxin has 20 kDa and an isoelectric point of pH 5.3, being most active at neutral pH [51]. Their mechanism of action is still not well known. However, KHS was proven to be a monomer that causes an increase in membrane permeability and the formation of ion channels. Ullivarri et al. [52] also described two chromosomal-encoded killer toxins, CF8 and M12, as highly thermostable, having an efficiency of 90% at an exposure to 90 °C, but also strongly influenced by pH, the range being 3.0–5.0 [52].

***P. membraniefaciens*** is a species that has been basically isolated from vegetative organs of plants and from fermentative processes. In a 2017 study, Belda et al. [53] isolated new strains of *P. membraniefaciens* from olive brines. Populations of halotolerant yeasts thrived in anthropogenic environments at pH values around 4.5 and at high temperatures. The presence of NaCl in concentrations comparable to concentrations in olive brine increased the killing character and the killing spectra of yeast isolates in this medium. Brine-isolated strains of *P. membranifaciens* were tested against a series of susceptible strains to establish if the killer phenotypes might be relevant for different biotechnological fields in which the exposure to stress condition is constant. The results showed the presence of at least two distinct phenotypes, confirming the idea that strains of the same yeast species can produce different toxins. Two strains, *P. membranifaciens* CYC 1106 and CYC 1086, were chosen due to their different spectrum of resistance and susceptibility to destruction, as well as being more active compared to the panel of yeasts that might be susceptible. Finally, the two *P. membranifaciens* strains produced two distinct toxins: PMKT (secreted by *P. membranifaciens* CYC 1106) and PMKT2 (secreted by *P. membranifaciens* CYC 1086) [53]. As a mechanism, PMKT has molecules of β1-6 D-glucan as primary receptors, and the Cwp2p protein as secondary receptors. The entire mechanism of cell binding is similar to K1 toxin from *S. cerevisiae*. Also, PMKT2 increases membrane permeability for H^+^; K^+^ or Na^+^ and can induce cell arrest in the S phase of the cell cycle.

**Figure 4:**
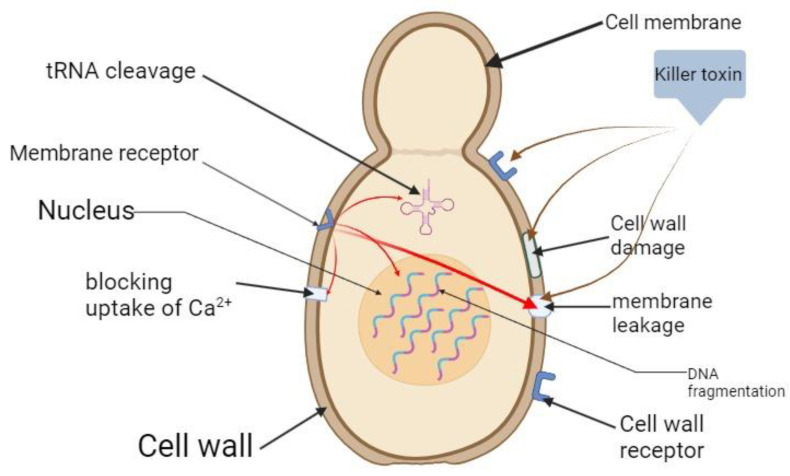
General mechanisms of antimicrobial action based on the synthesis of killer toxins.

Therefore, the way killer toxins act on potentially sensitive cells differs depending on the producing species and on their physical-chemical properties (Figure 4 and Table 2).

## 3. Red Yeasts

Some yeast species are known for their ability to accumulate carotenoids or similar chemical compounds during their growth. Therefore these yeasts are included in the generalized group called red yeasts or pigmented yeasts [54], comprising mainly members of the *Rhodotorula*, *Rhodosporidium*, *Sporobomlomyces* and *Sporidiobolus* genera, which are quickly identified based on their ability to form red-orange colonies when cultivated on agarized growth media [55]. Members of these genera have a worldwide distribution, being frequently isolated from air, soil, waters, plants’ surfaces and plants’ rhisospheres [56,57,58]. At the beginning, the scientific interest for the red yeasts group was mainly due to their ability to produce and accumulate various types of carotenoids. Lately, it was proven that the members possess some specific characteristics such as their capacity to assimilate and convert low-cost biomass (e.g., glycerol, lignocellulosic hydrolysates—wastes derived from the food and agro-industry, including wastewaters) into highly valuable compounds for circular bio economy [59]. Their ability to inhibit growth of undesired microorganisms is the research direction of maximum interest for the biomedical and agro-industrial fields. For this purpose, different microbial inhibition strategies, including the synthesis and secretion of compounds with antimicrobial action and the competition for nutrients from the environment, have been investigated for the members of the red yeast group [59,60,61,62].

### 3.1. Antimicrobial Compounds

The carotenoids are hydrophobic organic compounds included in the tetraterpenes group of natural compounds comprising eight isoprene units [63]. Their color can vary from yellow to orange or red. From a chemical point of view, they present very long-chain polyenes containing between 3 and 15 double bonds. Based on the presence or absence of oxygen atoms in their structure, the carotenoids are classified into xanthophylls and carotenes [64]. Among the best-known carotenoids produced by yeasts, the following stand out: β-carotene, torulene, torularhodin and astaxanthin [65]. The yeasts producing carotenoids belong mainly to *Phaffia*, *Rhodotorula*, *Sporobolomyces* and *Sporidiobolus* genera, and their carotenoids proved to be useful for the medical field (as antimicrobials agents, for the production of vitamin A, antioxidant compounds) but also in the industry food (as alternatives to food dyes obtained from chemical synthesis). *R. glutinis* and *R. mucilaginosa* are able to produce β-carotene, torularhodin and torulene using different industrial waste or polluting agents as growth substrate such as aromatic and aliphatic hydrocarbons, processed or unprocessed vegetable oils and glycerol [55]. Due to their high economic potential, the global market for these compounds is projected to reach USD 1.84 billion by 2027 with an compound annual growth rate of 3.4% for the forecast period [66]. Thus, searching for new methods of obtaining large quantities of carotenoids in a safe, sustainable and cost-effective manner represents a research direction with numerous economic implications. In this context, the red yeasts-based production of carotenoids offers a promising alternative which implies gaining more knowledge about their carotenogenic metabolism and the associated genes.

#### 3.1.1. Carotenoids Biosynthesis in Yeast Cells

**Figure 5:**
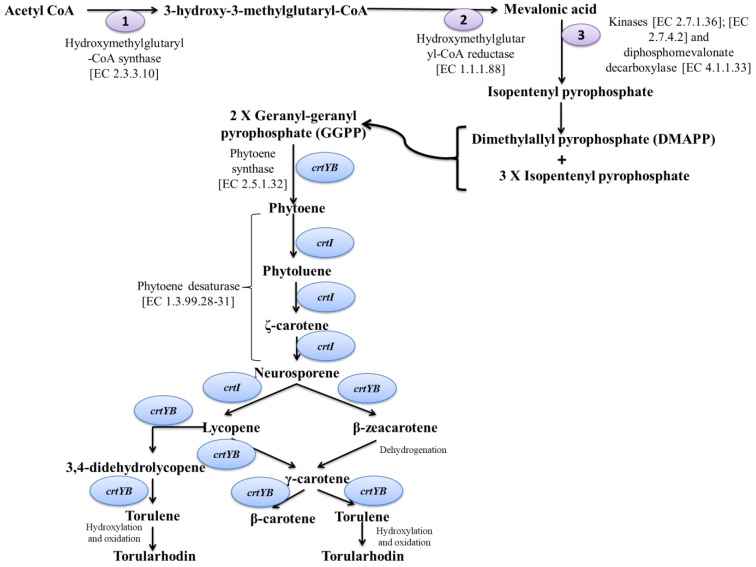
Torulene, torularhodin and β-carotene biosynthetic pathway and the encoded genes described for *Rhodotorula* strains (after [67,68]).

The general metabolism of carotenoids biosynthesis (Figure 5) in the yeast cell begins with the formation of the universal precursor isopentenyl pyrophosphate (IPP) and, subsequently, dimethylallyl pyrophosphate (DMAPP) through the mevalonate pathway (MVA) [56,67,68,69,70]. For other carotenoid-producing organisms, such as bacteria and plastids from plants, the first stage of terpenes production is associated with another metabolic pathway entitled the MEP biosynthesis pathway, which involves obtaining IPP and DMAPP through a series of chemical reactions starting with pyruvate and glyceraldehyde-3-phosphate condensation to form 1-deoxy-D-xylulose-5-phosphate, which is further reduced to form 2-C-methyl-d-erythritol-4-phosphate (MEP). Subsequently, MEP is phosphorylated and undergoes cyclization reaction to produce, in the end, IPP and DMAPP [70].

Many studies have been conducted in order to determine the role of different genes in the carotenoids’ biosynthesis. Landolfo et al. [71], based on the functional annotation of the *R. mucilaginosa* C2.5T1 genome, proved that the *HMG1* gene encodes the HMG-CoA reductase, while *ERG12* codes the mevalonate kinase. These genes are not exclusively involved in the caratonoids’ biosynthetic pathway, but their expression is also highly induced during the exponential growth phase and due to the presence of mevalonate precursors. Moreover, a cluster was identified composed of three genes that encode enzymes: *crtB*, which encodes phytoene synthase; *crtY*, which encodes lycopene cyclase, and *crtI*—phytoene desaturase. Genes encoding geranylpyrophosphate synthase, carotenoid oxygenase (*crtX*) and phytoene synthase/lycopene cyclase (*crtYB*) are not found in the same cluster; however, they are located nearby and are convergent transcribed versus the cluster [67,72].

##### Role of Carotenoids in the Yeast Cell

In general, the aerobic growth of yeasts involves exposure to reactive oxygen species (ROS), which, in high concentrations, can cause alterations at the DNA molecule, protein or lipids from the cell membranes structure [73]. Maintenance of redox potential in the yeast cell following exposure to stress oxidative is achieved by limiting the accumulation of ROS, controlling metabolism iron/copper and activation of thiol-reductase systems (thioredoxin and glutathione) [74]. The main molecular mechanisms involved in resistance to oxidative stress have been described for *S. cerevisiae*, but most yeast species show basically similar mechanisms. The signaling pathway involved shares common ground with the HOG cascade, with the major sensors of oxidative stress being Sln1 and Sho1 receptors. In this case, however, the central element of the path of signaling involved in the cellular response to oxidative stress is the protein Yap1. This protein belongs to the highly conserved group of transcription factors called AP-1 and which share the DNA-binding zinc-finger domain. Yap1 binds to the site of YRE recognition (5′-TT/GAC/GTAA-3′) from the promoter structure of some target genes such as *TRX2* (encoding thioredoxin), *GSH1* (encoding γ-glutamylcysteine synthase), *GSH2* (encodes glutathione synthase 2), *TRR1* (thioredoxin reductase 1), *GPX2* (glutathione peroxidase 2), *TSA1* (thioredoxin peroxidase 1) and *AHP1* (alkylhydroperoxide reductase 1) [73,75]. The elimination of ROS is achieved by the synergistic action of antioxidants produced as result of the binding of the YAP1 factor to the YRE, namely superoxide dismutases, catalases, thioredoxin and glutathione. The splitting of H_2_O_2_ to O_2_ is accomplished by the catalytic action of two catalases located in the cytoplasm, respectively, in peroxisomes. Removal of O_2_^−^ supposes the intervention of some cytosolic superoxide dismutases and a superoxide dismutase mitochondrial (which mainly intervenes in the removal of O_2_^−^, resulting from oxidative phosphorylation) [76,77].

The carotenoids play essential roles in the yeast cells since they are membrane-protective antioxidants involved in scavenging the singlet molecular oxygen and peroxyl radicals.

Moline et al. [78] proved that the pigment assimilation in the yeast cells is associated with enhanced survival to UV-B exposure. By comparing different strains of *R. mucilaginosa*, including two mutant strains (one a hyper-carotenoid-producing strain and the other one colorless) exposed to UV-B radiation in presence or absence of a carotenogenic inhibitor, diphenylamine, the authors showed that the hyper-productive strain presented an enhanced survival growth rate (up to 250%) compared to the parental strain. However, no direct correlation was observed when analyzing the DNA damage caused by UV-B exposure. More than that, some yeast species, including red yeast species, are able to produce mycosporines, intracellular colorless photoprotective compounds that act as sunscreen protective compounds against UV radiation. Thus, their mechanism of protection against oxidative stress is highly efficient and of great interest for antioxidant research direction [79,80].

#### 3.1.2. Main Classes of Carotenoids and Their Role in the Biomedical Field

- *β-carotene* is an isoprenoid compound comprising two β-ionone rings connected by a polyene chain with nine conjugated double bonds. This compound exhibits highest absorbance at 450 nm and is currently being investigated due to its potential for biomedicine being the main source of vitamin A in the human diet [81]. Moreover, due to the presence of the ionone rings, the β-carotene exhibits high antioxidant activity and can be used as a lipid scavenger with numerous applications in cardiovascular disease, macular degeneration and immune response stimulation [82,83,84]. Furthermore, this pigment is soluble in edible oil providing yellow to red colors, therefore being currently used in the food additives industry as natural food colorant [85,86,87,88]. Among yeast species, *Phaffia rhodozyma* is well known as being able to produce and accumulate β-carotene due to its ability to express enzymes involved in the metabolic pathway of carotenoids’ synthesis such as isopentenyl pyrophosphate isomerase (IDI), geranylgeranyl diphospate synthase (CRTE), phytoene synthase (CRTB), pythoene desaturase (CRTI) and lycopene cyclase (CRTY) [85].
- *Astaxanthin* is another type of carotenoid described for yeasts species. Chemically, it belongs to the group of xanthophylls and presents two polar β-ionone rings with one hydroxyl and ketone group, connected by a non-polar chain. Its high antioxidant properties are determined by the presence of 13 double bonds, and its polar character is determined by the presence of hydroxyl and ketone groups attached to the β-ionone rings. Apart from its antioxidant properties, the astaxanthin is currently being investigated for its anti-inflammatory and anti-apoptotic functions with high potential for preventing cardiovascular diseases [89], for improving the neurologic functions after brain injuries [90], as renoprotective agents after exposure to lithium [91] and even for reversing the cigarette smoking-induced oxidative stress and inflammation [92]. The current assessments regarding the evolution of astaxanthin market showed that was expected to reach over USD 3500 million by 2023 [93]. Although highly valuable for mankind, the astaxanthin is in present produced mainly throughout chemical synthesis, and the price per kilo exceeds USD 2000 [94,95]. In case of yeasts, *P. rhodozyma* (*Xanthophyllomyces dendrorhous*) is known for its ability to produce 3R-3R′ enantiomer in a free form, with the main disadvantage of a low yield of accumulation (about 300 µg/g), which is far from being cost-effective [94]. The production of astaxanthin in the *P. rhodozyma* cells requires two additional reactions involved in the transformation of β-carotene into several intermediates first by incorporating two 4-keto groups through a reaction catalyzed by a ketolase following by the addition of two 30 hydroxy groups, a reaction mediated by a hydroxylase. For *P. rhodozyma*, both reactions are mediated by an astaxanthin synthetase, CrtS encoded by the *crtS* gene [96]. Even so, the activity of this enzyme is influenced by the presence of another enzyme, a cytochrome P450 reductase encoded by the *crtR* gene, which is responsible with providing the necessary electrons for the oxygenation reactions [97]. The main strategies approached to increase astaxanthin production yield in yeasts consist in exposure to non-specific mutagenesis processes using N-Methyl-N′-nitro-N-nitrosoguanidine, UV light or low-dose gamma irradiation [98,99]. Even so, random mutagenesis is associated with the decrease in biomass yields and growth rates, thus being thus hard to determine the positive impact on the cost-effective production of astaxanthin [100]. Nevertheless, *P. rhodozyma* remains one of the most important microorganism to be studied for the production of this type of carotenoid [95].
- *Torulene and torularhodin* are composed of one β-ionone ring with a polyene chain with 12 conjugated double bonds [65], but in the case of the latest, an additional carboxyl group at the end of the polyene chain is described, thus bearing a higher oxidation state [101]. Although intensively studied during the last decade, the impact of these carotenoids on human body is not well understood due to their absence in food. At present, there are some studies concerning their impact on rats lungs, liver and kidney function when administrated as food additives and no significant effect was observed [102,103]. Also, these carotenoids showed protective properties against neoplastic liver changes induced by dimethyl nitrosamine in the case of mice and inhibited the development of prostate cancer [104,105]. The production of torulene and torularhodin was determined in various cultivation condition of different strains belonging to the *R. glutinis*, *R. mucilaginosa* (*R. rubra*), *R. graminis*, *S. salmonicolor*, *S. pararoseus*, *S. johnsonii* and *S. ruberrimus* species [68]. High amounts of torulene and torularhodin were obtained in the case of *S. ruberrimus* cultivated in the presence of raw glycerin as carbon source (70 mg/L respectively, 350 mg/L) [106]; *S. salmonicolor* cultivated in the presence of saccharose (273.7 μg/g dry biomass respectively, 458.3 μg/g dry biomass) [107]; and *R. glutinis* cultivated with white-light irradiation (32.2 mg/100 g cells dry weight, respectively, 14.2 mg/100 g cells dry weight) [108]. Although the general biosynthesis pathway of torulene and torularhodin is presented above, there are some species, such as *S. pararoseus* or *Neuraspora crassa*, in which these compounds can also be produced from 3,4-dehydrolycopene, an intermediate compound formed from lycopene under the enzymatic action of a phytoene desaturase. Subsequently, the intermediate is transformed into torulene through a reaction catalyzed by phytoene synthase/lycopene cyclase AL-2 [109].

#### 3.1.3. Antimicrobial Activity of Carotenoids

Apart from their antioxidant properties, the carotenoids produced by the red yeasts have been studied for their antimicrobial potential, including antibacterial and antifungal assessments both as crude extracts or purified compounds. According to Table 3, many red yeast extracts have shown strong antimicrobial activity against a wide range of pathogenic and potential pathogenic bacteria and fungi.

In addition, due to their high biomedical potential based on both their antioxidant and antimicrobial activity, the carotenoids from yeasts have also been used for the production of implanted medical products. Titanium implants dipped in torularhodin solution of 5 mg/mL inhibit the growth of Gram-negative bacteria such as *E. coli* ATCC8738, *P. aeruginosa* ATCC9027 or Gram-positive bacteria—*S. aureus* ATCC 25923, *E. faecalis* ATCC29212, *B. subtilis* ATCC 6633—and also inhibit their biofilm-forming capacity [118]. Members of red yeasts group were successfully used for the synthesis of metal nanoparticles. Zaharan et al. [119] used a *R. glutinis* strain to prepare silver nanoparticles with diameters ranging from 2.5 to 20 nm, while Soliman et al. [120] proved that the *Rhodotorula* sp. strain ATL72 from salt marches near the Mediterranean Sea, Egypt, could be successfully used for the production of oval-shaped silver nanoparticles with diameters ranging from 8.8 to 21.4 nm with strong antimicrobial activity against *Streptococcus* sp., *Bacillus* sp., *Staphylococcus* sp., *Shigella* sp., *E. coli*, *P. aeruginosa*, *Klebsiella* sp. and *Candida* sp.

### 3.2. Competition for the Nutritive Substrate

Apart from the synthesis of antimicrobial compounds, the red yeasts also exhibit antagonistic characteristics attributed to the competition for nutrients. This mechanism is probably the most important factor in yeast ecology, in general, due to their wide spread in different ecological niches where the yeasts have developed complex mechanisms aimed to inhibit the competing microorganisms [121]. The mechanisms is highly valuable for the development of biocontrol strategies to protect fruits against fungal contamination [122]. Damaging the fruits’ surface as a result of different injuries acquired during harvesting, transportation or storage causes an ideal environment for necrotrophic pathogens’ contamination. At the site of the lesion, high quantities of nutrients such as glucose or other sugars become highly available for pathogens, stimulating their growth. Although the plants present some mechanisms of protection in these circumstances, such as those aiming to avoid pathogen invasion (accelerating wound healing process, synthesis of phenolic compounds, activating the jasmonate signaling pathway), the response is not fast or efficient enough [123,124]. Thus, the contribution of other microorganisms as protective agents against pathogens infections can be decisive.

In the case of the yeasts, the first mechanism of antagonistic activity described is sugar competition. Due to their highly versatile metabolism, the yeasts can use as carbon source a wide range of carbohydrates including disaccharides and monosaccharides. As an example, the red yeast *S. roseus* inhibits the growth of *B. cinerea*, a plant pathogen that causes many agricultural loses due to its high resistance to classical fungicides used in the current agricultural practices [125], by limiting its access to glucose and blocking the conidial germination [126]. A similar effect was observed in the case of a *R. mucilaginosa* strain isolated from the surface of apple fruits. The yeast cells attached directly to the B. cinerea spores and hyphae through proteins found in the yeast cell wall [127]. Also, the same strain exhibited an inhibitory effect against *P. expansum* through a mechanism which is not based on calcium deprivation [128]. Another red yeast, *R. paludigenum*, isolated from a marine environment, showed important antifungal activity based on nutrient competition against *A. alternata* tested both using in vitro and in vivo on cherry tomato, frequently affected by postharvest black rot disease [129]. In the case of *R. fluviale*, the main mechanism of antifungal activity against *B. cinerea*, which colonize apple fruit, is based both on the competition for the space and the induction of the β-1,3-glucanase production in apple tissue [130]. A study conducted by Huang et al. [131] proved that N-acetylglucosamine exposure of *R. paludigenum* enhances its antifungal activity against *P. expansum* when tested directly on pears. The presence of N-acetylglucosamine boosts the viability of the yeast strain under stress conditions and activates the expression of antioxidant-related enzymes coding genes such as those coding peroxisomal catalase, thioredoxin reductase, gluthation peroxidase, glutathione reductase and superoxide dismutase. Another important aspect when discussing the potential use of microbial strains as biocontrol agents is their adaptability to low temperatures and controlled atmosphere conditions associated with the fruit storage conditions. According to Guozheng et al. [132], a *R. glutinis* strain isolated from apple fruits exhibited a higher protective role against *A. alternata-*, *P. expansum-*, *B. cinerea-* and *R. stolonifer*-contaminated sweet cherries under controlled atmospheres with 10% O_2_ +10% CO_2_ incubation for 60 days. Their enhanced potential was compared by testing their protective role on fruits stored at 0 °C for 30 days.

**Figure 6:**
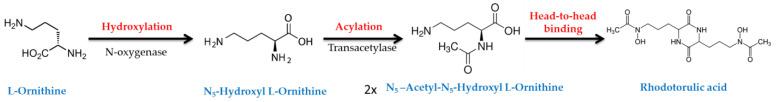
Chemical transformation of L-ornithine in rhodotorulic acid [137,138,139].

Another nutrient competition mechanism is the uptake of iron, which is essential for microbial growth and, in some circumstances, for the expression of the pathogenic profile [133]. Under aerobic cultivation, iron forms insoluble ferric hydroxide complexes which causes a severe restriction of its bioavailability [134]. Some microbial species, including red yeasts, can secrete siderophores, iron-scavenging molecules through which the producing cell immobilizes iron from the environment for its own consumption. First, this mechanism was described for *M. pulcherrima*, which is able to produce and secrete a red pigment named pulcherrimin [135], but in the last decades, a similar mechanism was identified also in the case of red yeasts. Members of the *Rhodotorula* genera have shown important antimicrobial activity based on iron competition, mainly against phytopatogenic fungi. *Rhodotorula* strains are able to produce rhodotorulic acid, a siderophore synthetized starting from the non-proteinogenic aminoacid L-ornithine, which is hydroxylated and acylated at its δ-amino group [136], forming N5-acetyl-N5 hydroxy-L-ornithine. Then, two molecules of N5-acetyl-N5 hydroxy-L-ornithine are linked head-to-head to form a diletopiperazine ring [137] (Figure 6).

The rhodotorulic acid is relevant for the biologic control of postharvest disease caused by *P. expansum*. Its production by *Rhodotorula* cells is stimulated by the presence of ornithine [136], sucrose and ammonium acetate. On the contrary, in the presence of glutamate, other saccharides than the sucrose of a carbon source, have no significant impact on its production [140]. Urea is another chemical compound that stimulates the production of rhodotorulic acid, mainly due to the fact that it is a metabolite related to the basic amino acids ornithine and arginine [136,141]. Although rhodotorulic acid has been known since the beginning of the 1970s [142], there are yet numerous questions to be answered regarding the uptake of the iron after the secretion of rhodotorulic acid into the environment or the genetic support for the entire biosynthesis pathway within the most efficient red yeast species. Until answers are found, rhodotorulic acid remains an antimicrobial strategy with massive potential to combat phytopathogens such as *B. cinerea* or *P. expansum* that affect agricultural crops before or after harvest and storage.

## 4. Biosurfactants

During last decades, there has been a growing interest in using ecofriendly compounds for inhibiting the growth and multiplication of pathogenic microorganisms, both for the protection of crops and for combating the human and animal disease. The biosurfactants, extracellular compounds produced by microorganisms (yeast, fungi and bacteria), represent an interesting alternative for the chemical products used in biocontrol as fungicides or in biomedicine as antimicrobial agents.

The biosurfactants are amphiphilic molecules, with a hydrophobic (consisting in fatty acids or acetyl groups) and a hydrophilic moiety (represented by carbohydrates or proteins), able to lower the surface tension between two immiscible fluids, thus facilitating the assimilation of hydrophobic substrates in the microbial cells. The variability of their chemical structure and the ability to form micelles at the CMC (critical micelle concentration), along with their high biodegradability and stability at various temperature, pH and salinity values, are responsible for their large range of practical applications. In this respect, the biosurfactants can be classified according to their molecular weight in low-mass biosurfactants (glycolipids, phospholipids and lipopeptides), used for lowering the surface and interfacial tension, and high-molecular mass biosurfactants (lipoproteins, lipopolysaccharides or polysaccharides-protein complexes) with good ability to stabilize oil-water emulsions [143,144].

The biosurfactants produced by yeasts receive high consideration due, on one hand, to the restricted access of bacterial biosurfactants in food and therapeutics, and, on the other hand, to the fact that many biosurfactant producing yeast species are presently recognized as safe (GRAS) and do not produce biocompounds and extracellular proteins harmful for human and animal use. The biosurfactants produced by the yeast species belonging to the *Candida*, *Pseudozyma*, *Saccharomyces*, *Rhodotorula*, *Yarrowia*, *Kluyveromyces*, *Wickerhamomyces* and *Debaryomyces* genera are classified according to their chemical structure in: glycolipids (sophorolipids, mannosylerytrithol lipids), polymeric biosurfactants (carbohydrate-protein complexes, carbohydrate-protein-lipid complexes, mannan-lipid-proteins, lipomanan, liposan), fatty acids and lipids [145,146].

### 4.1. Genes and Regulation

Due to their specificity of action and their different chemical structure, the biosurfactants have a wide range of biotechnological applications. Nevertheless, the glycolypids (sophorolipids—SLs and mannosylerytrithol lipids—MELs) are the most studied classes of yeast biosurfactants, with important antimicrobial activity, used in biocontrol and therapeutics.

### 4.2. Sophorolipids

The sophorolipids (SLs), produced mainly by strains belonging to *Candida (Pseudozyma) bombicola*, *Candida apicola*, *Candida stellata*, *Rhodotorula babjevae*, *Debaryomyces hansenii* and *Wickerhamiella domercqiae* species, have a hydrophilic moiety represented by a sophorose unit and a hydrophobic moiety consisting of fatty acids, with different lengths, degrees of unsaturation and acetylations corresponding to two types of sophorolipids: acidic (with a free carboxylic end) and, respectively, lactonic (internally esterified at the 4″, 6′ or 6″ position). The best-studied SLs are those produced by some strains belonging to the *Candida (Starmerella) bombicola* species. The yeast can assimilate n-alkanes or tryglicerides, which are metabolized subsequently into (i) fatty acids (process catalysed by citochrome P450monooxygenase, respectively, by lipases), (ii) hydroxy fatty acids (citochrome P450monooxygenase), (iii) glucolipids (glucosyltransferase I), (iv) sophorolipids (acidic form) (glucosyltransferase II) and, from this point, (v) either into acetylated acidic sophorilipids (acetyltransferase) or (v’) into lactonized sophorolipids (lactonesterase) and, finally, into acetylated lactonized sophorilipids (acetyltransferase) [147]. During the National Belgian IWT project “Biosurf”, Van Bogaert et al. [148] reported the identification of a gene cluster of about 13,000 bp, consisting of five genes involved in SL synthesis in *S. bombicola*, codifying a cytochrome P450 monooxygenase with function of fatty acids hydroxylase (*cyp52m1*), two glucosyltransferases I and II (*ugta1*, respectively, *ugtb1*), an acetyltransferase (*at*) and a transmembrane transporter protein (mdr). Later, Ciesielka et al. [149] identified the gene *sble* placed about 2500 bp upstream of the cluster, encoding a lactone esterase involved in sophorolipid lactonisation.

Several studies were developed aiming the elucidation of regulation of SL synthesis in *S. bombicola*. Thus, the knock-out mutant strains for the gene *MFE2* encoding an enzyme with double function (3-hydroxyacyl-CoA dehydrogenase and enoyl-CoA hydratase) in the β-oxidation pathway of fatty acids in the peroxisomes, presented a 1.7–2.9 higher production of sophorolipids compared with the wild type strain [150]. Recently, Liu et al. [151] showed that the Rlp protein, sharing a 30.5% identity and 55.4% aminoacid sequence similarity with the membrane protein Rim9 from *Yarrowia lipolytica*, and two zinc-knuckle type transcription factors Ztf1 and Leu3, play important roles in the regulation of sophorolipid synthesis. Thus, the ΔrlpΔleu3Δztf1 strains exhibited an increased sophorolipid production (up to 50.51%) compared to the parental strain. The deletions also upregulated the activity of key enzymes involved in: Δztf1—transformation of sucrose to glucose (furanglycosidase) and transition of oxalacetic acid to phosphoenolpyruvate (phosphoenolpyruvate carboxykinase); Δleu3—transformation of Acetyl CoA to fatty acids; Δrlp—transformation of hydroxy fatty acids to acid SL (glucosyltransferases I and II), respectively, transformation of acetylated acid SL to acetylated lactonic SL (lactone esterase). Proteomics studies revealed that heme binding resistance protein 1 (DAP1) might be involved in cytochrome P450 monooxygenase regulation [152]. On the other hand, bioreactor experiments on *S. bombicola* [153] and flask experiments on *W. domercquiae* [154] showed that the presence of magnesium ions upregulated the activity of sble gene favoring the formation of lactonic SLs, while the iron ions downregulated the sble gene, leading to the synthesis of acidic SLs.

### 4.3. Mannosylerytrithol Lipids

The mannosylerytrithol lipids (MELs) are glycolypid biosurfactants produced by yeast strains from the *Pseudozyma* species (*P. antarctica*, *P. hubeiensis*, *P. rugulosa*, *P. tsukubaiensis*, *P. crassa*). The hydrophilic moiety is represented by 4-O-β-D-mannopyranosyl-meso-erythritol formed by a mannose molecule linked to erytrithol, while the hydrophobic part consists in fatty acids and/or acetyl groups. Depending on the degree of acetylation of the mannosylerytrithol component, the MELs are classified in: MEL-A—diacetylated at C4 and C6, MEL-B—monoacetylated at C4 or MEL-C—monoacetylated at C6 and MEL-D—nonacetylated. It seems that the structure of MELs is species related, with *P. antarctica* producing a combination of MEL-A, B and C, *P. parantarctica*, *P. fusiformata*, *P. crassa*—MEL-A and *P. tsukubaiensis*—MEL-B [155,156]. The biosynthetic pathway of MEL described in *P. antarctica* begins with the assimilation of n-alkanes, fatty alcohols or fatty acids with C_12_–C_18_, which are degraded to corresponding Acyl-CoA, which is further involved in four metabolic processes: (i) β-oxidation with formation of Acetyl-CoA; (ii) chain shortening pathway (C_12_–C_18_ substrates gave MELs with Cn-2 fatty acids); (iii) chain elongation; (iv) intact incorporation. While the last two pathways lead to synthesis of cellular lipids, the first leads to de novo synthesis MELs via erythritol, mannosylerytrithol, MEL-D and, finally, MEL-A, B or/and C [157]. The genes involved in this metabolic pathway and described for *P. antarctica*, *P. tsukubaiensis*, *P. aphidis* and *P. graminicola* form a cluster, in a species-specific order and encode an erythritol/mannose transferase (*Emt1*), two acyltransferases (*Mac1* and *Mac2*) and an acetyltransferase (*Mat1*) [156,158,159]. In the cluster, the gene *MMF1*, encoding a plasma transporter protein, is also present [156,159]. Additional studies have revealed that the erythritol/mannose transferase encoded by the gene *PtEMT1* from *P. tsukubaiensis* is also responsible for catalyzing the sugar conformation of MELs [160], while the ΔPgEMT1 *P. graminicola* mutant cells were significantly smaller in size and the colonies were glossy, with an intense yellow color [159]. The studies aimed to elucidate the regulation of MEL synthesis in the same species, *P. graminicola*, showed that the genes from the MEL cluster are not expressed as a block, their activity is not induced by the fatty acids and that the gti1 and pac2 transcription factors (members of the WOPR family) upregulate MEL-C synthesis [159]. In *P. antarctica*, overexpression of lipase gene *PaLIPA* was found responsible for higher rates of oil assimilation and MEL-B production [161]. In fact, previous transcriptomic studies of the strain *P. antarctica* T-34 showed that, in presence of vegetable oils as carbon substrate, the cell metabolism suffers modifications aimed to assure enhanced extracellular MEL synthesis [162].

#### 4.3.1. Biosurfactants for Biocontrol

The biosurfactants have a wide range of applications in agriculture, ranging from bioremediation of agricultural soils polluted with hydrocarbons or heavy metals to biostimulation of plant growth and biocontrol of phytopathogens which affect crops. At present, most agricultural practices use chemical pesticides, sprayed directly on the surface of plants, or used as solution for dipping the fruits. In order to increase the effectiveness of the process, chemical surfactants are mixed with the pesticides as adjuvants. On the contrary, the biosurfactants can be directly applied on the surface of the plants, assuring the increase of antimicrobial activity of various microorganisms or biocompounds [163]. Thus, in the bioformulations for biocontrol, the biosurfactants can be: (i) mixed with cultures of various microorganisms with known antagonistic activity or with various antifungal compounds, (ii) encapsulated in liposomes or nanoparticles for enhanced stability or (iii) used as biocompounds produced by co-cultures growing on the surface of the plants [164].

##### Sophorolipids as Biocontrol Agents

**Figure 7:**
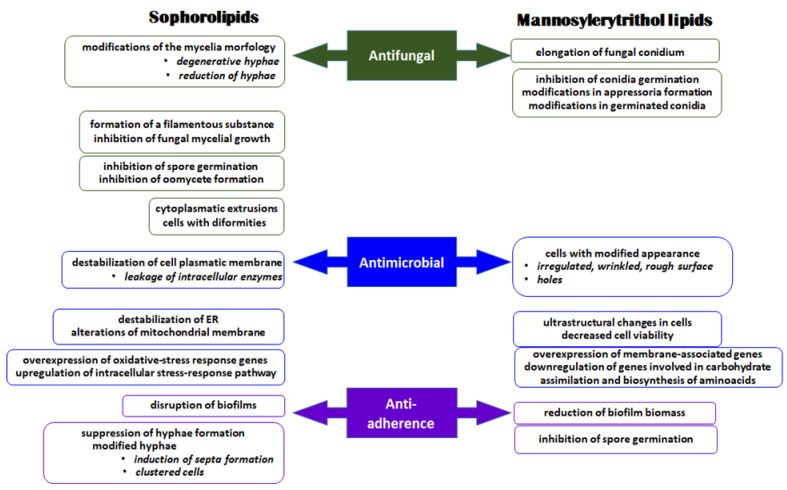
The main mechanisms of antimicrobial activity of sophorolipids and mannosylerytrithol lipids.

Until present, sophorolipids produced by *S. bombicola*, *Rhodotorula* species (*R. babjevae*, *R. glutinis*) and *W. domercqiae* have been described as promising biocontrol agents (Figure 7).

The SL produced by the strain *S. bombicola* NRLL Y-17069 mixed (in final concentration of 1%) with phosphinothricin (a water-soluble herbicide) (concentration 1 mg/mL) determined important reduction of both dry and fresh weight of the weed *Senna obtusifolia* (sicklepod) and lowered the herbicide damage rate (HDR) after 10 days of treatment [165]. During another study, the SL produced by *S. bombicola* ATCC 22214 (in concentration of 1800 mg/mL) determined the inhibition of fungal mycelial growth of *A. flavus*, *Aspergillus melleus*, *Aspergillus ochraceus*, *Aspergillus parasiticus*, *A. niger*, *F. oxysporum*, *B. cinerea* and *Rhizopus* spp., with the best results against *A. ochraceus* (67%), *B. cinerea* (50%) and *A. melleus* (47%). Moreover, the scanning electronic microscopy (SEM) analysis revealed that the SL determined modifications of the morfology of *F. oxysporum*, *A. niger*, *B. cinerea* and *Rhizopus* spp. mycelia, most probably due to the interruption of the phospholipid layer of the cell membrane [166]. The same SL biosurfactant (in concentration of 2 mg/mL) determined the inhibition of mycelial growth of *Pythium ultimum* (95%), *B. cinerea* (75.7%), *Sclerotium rolfsii* (64.3%) and *Rhizoctonia solani* (28.5%) in Petri plates [167]. The SEM images showed cells with deformities (*B. cinerea*), inhibition of oomycete growth and reduction of hyphae (*P. ultimum*), degenerative hyphae (*R. solani*) or the formation of a filamentous substance (*S. rolfsii*). The SL was reported as causing not only changes in cell membrane permeability but also cytoplasmatic extrusions and an inhibition of enzymes involved in hyphae formation. These mechanisms were also mentioned by Chen et al. [154], who observed that the SL produced by *W. domercqiae* Y_2A_ inhibited the mycelial growth and spore germination and led to formation of hyphae with morfological changes for Fusarium sp., *F. oxysporum*, *Fusarium concentricum*, *Pythium ultimum*, *Pyricularia oryzae*, *Rhizoctorzia solani*, *Alternaria kikuchiana*, *Gaeumannomyces graminis* var. tritici and *Phytophthora infestans*. Moreover, the β-glucosidase activity in *P. infestans* mycelia was reduced from 2.53 U (in control) to 0.12 U (by adding 0.5 mg/mL of SL). The SL from *S. bombicola* ATCC 22214 was also effective in preventive-curative treatment against *B. cinerea* (i) directly sprayed (in concentration of 1 mg/mL) on the tomato leaves, respectively, or (ii) by dipping the tomatoes in SL solution (4 mg/mL) [167]. Sen et al. [168] reported a new SL biosurfactant obtained from *R. babjevae* YS3 with significant antifungal activity against *Colletotrichum gloeosporioides*. Another biosurfactant produced by a *R. glutinis* strain showed antifungal activity against *A. niger*, *Alternaria* sp., *Rhizopus* sp. and *Syncephalastrum* sp. [169]. SLs were also used as adjuvants to chemical fungicides such as epoxiconazole, sulfur and rimsulforon, enhancing (up to 99%) their antifungal activity against *Blumeria graminins* f. sp *hordei* [170]. A formulation containing 100–500 g SL/ha was recommended for improving biomass formation and development the harvestable part of plants [171].

##### Mannosylerytrithol Lipids as Biocontrol Agents

There are few reports concerning the use of MEL in agriculture. Thus, a purified (95%) MEL-B solution in concentration of 158 mg/L stimulated *Letuca sativa* L. seed germination, growth and seedling development [172]. The same concentration biostimulated the formation of lateral roots, without any phytotoxic effect. Determinations of enzymatic activities showed that a higher MEL-B concentration (632 mg/L) increased peroxidase activity during the first three days of germination, while the activity of polyphenol oxidase was enhanced until the fifth day of germination. The wetting ability of MELs from *P. antarctica* has been investigated in order to develop new strategies for their use in agriculture. Fukuoka et al. [173] showed that a MEL solution (0.1%) had the ability to spread evenly on the surface of Gramineae plant, while a pretreatment with 0.08% (*v*/*v*) solution of MEL enhanced the fixation of *Bacillus subtilis* cells on the surface of leaves, thus proving the potential application of MELs as agrospreaders. Studies regarding the antifungal activity of MEL forms produced by different *Pseudozyma* species have proved that MEL-A suppressed the germination of *Blumeria graminis* f. sp. tritici conidia, MEL-C affected Colletotrichum dematium conidia and MEL-B was effective against both *C. dematium* and Magnaporthe grisea conidia, while the application of MEL solutions on the surface of wheat leaves and inhibited powdery mildew appearance caused by *Blumeria graminis* f. sp. *tritici* [173]. Other effects were also reported, such as elongation of the fungal conidium or modifications in appressoria formation in germinated conidia, mainly caused by the hydrophobicity of the biosurfactant (Table 4).

#### 4.3.2. Biosurfactants in Biomedicine

In addition to their importance for bioremediation and various industry domains (cosmetics, food, oil), the biosurfactants have a wide range of biomedical applications as antimicrobial and anti-adherent agents, antioxidants, adjuvants for pharmaceutical products, antitumoral and anti-inflamatory compounds or in wound healing processes (Figure 7).

##### Sophorolipids for Biomedical Use

SLs and SL-type biosurfactants produced by different yeast species have been described as having antimicrobial and antibiofilm activities based on various mechanisms. Thus, a SL produced by *S. bombicola* have spermicidal properties and is cytotoxic against human immunodeficiency virus (HIV), a fact most probably related to the composition of their hydrophobic and hydrophilic moieties, hence their ability to form micelles which destabilize the plasma membrane of the cells, alter the hydrophobicity and the cell surface charge [174,175]. The process might represent the basis for the development of studies concerning the use of biosurfactants for the treatment of cancer. The same mechanism was mentioned by Kim et al. [176], who proved that SL produced by the strain *C. bombicola* ATCC 22214 had antimicrobial activity against bacterial strains of *B. subtilis*, *Staphylococcus xylosus*, *Streptococcus mutans* and *Propionibacterium acne* at concentrations from 0.5 to 4 ppm, causing an enhanced leakage of intracellular enzymes mediated by SL–cell membrane interactions. During the same study, it was found that the lactonic SL form exhibited higher antimicrobial activity than the acidic SL form. Another study showed that SLs produced by *C. bombicola* and *C. apicola* used in concentration of 29 mg/L, inhibited the growth of bacterial strains from *B. subtilis*, *S. epidermidis* and *Streptococcus faecium* species [177].

Other glycolipid biosurfactants produced by yeasts are also efficient against drug-resistant pathogenic microorganisms. For example, a SL-type biosurfactant produced by *D. hansenii* MK9 and a rhamnolipid-type biosurfactant from *R. mucilaginosa* SP6, used in a concentrations of 2 g/L, had high antimicrobial activity against bacterial and fungal human pathogenic strains belonging to *Proteus vulgaris*, *E. coli*, *K. pneumoniae*, *S. aureus*, *Micrococcus luteus*, *C. neoformans*, *C. albicans* and *A. niger* [178]. Moreover, the study showed that the biosurfactants had lower impact on multi-drug resistant pathogens and, respectively, on Gram-negative bacteria compared to Gram-positive bacteria, most probably due to their different cell wall structure.

The SLs can also affect the ROS mediated endoplasmatic reticulum stress mechanism and the mitochondrial functions in *C. albicans* cells [179]. Thus, *C. albicans* cells treated with a SL from *S. bombicola* MTCC 1910 presented high levels of expression of *SOD* and *CAT1* genes involved in an oxidative stress response, which enhanced the release of Ca^2+^ ions from the endoplasmatic reticulum (ER), causing alterations of the mitochondrial membrane potential. The processes were confirmed by assays showing the upregulation of *HAC1* gene (a stress-response marker for ER) and the activation of the HOG-MAPK stress-response pathway.

The SLs are also known as having antibiofilm formation potential. Thus, the SLs are more effective against planktonic *C. albicans* cells compared to matured biofilm composed by dense hyphae. In the presence of SLs, the biofilm suffers changes due to loss of cell membrane integrity, a fact indicated by the presence of cells with perforated outer membranes [180]. Hyphal growth inhibition (up to 55% using 15 μg/mL SL) might be related to downregulation of *ASP3* and *HPW1* genes (involved in adhesion to the substrate), as shown by an analysis of the transcript levels of these genes. Similar results were obtained in an experiment using an SL produced by *Starmerella riodocensis*. The biosurfactant, a combination of acidic and lactonic SL forms, showed activity against *C. albicans* biofilm formation, with the SEM analysis revealing the fact that longer exposure to SL not only suppresses hyphae formation but also induces septa formation and the appearance of cells with bud scars (for SL treated cells after 90 min or 24 h after adhesion) [181].

SLs have also been reported as producing disruption of biofilms of *E. coli* and *B. subtilis*; in the case of the last one, the analysis also revealed individual or small clusters of cells as well as cells with damaged membranes [182].

#### 4.3.3. Mannosylerytrithol Lipids for Biomedical Use

The studies developed during last two decades have emphasized the impressive potential of MELs for therapeutic use, as antimicrobial and/or anti-adhesive agents, as well as potential carriers and adjuvants in cancer treatment. Thus, a MEL-A biosurfactant produced by *Pseudozyma aphidis* was reported to determine *B. cereus* cell deterioration (appearance of irregulated, wrinkled cells), to inhibit spore germination and cell multiplication and to cause cell membrane damage leading to leakage of nucleic acids (DNA) and proteins [183]. The same biosurfactant was further investigated against *S. aureus* ATCC 6538 and proved to induce cell growth arrest within 10 h at 2 × MIC (minimum inhibitory concentration—the lowest concentration of antimicrobial agent), with the appearance of modified cells with holes, ultrastructural changes and late apoptosis cell in a proportion of 50% at 2 × MIC, respectively, to reduce cell survival in the *S. aureus* biofilm formed on glass and stainless steel [184]. Another study using the same MEL-A [185] demonstrated its antimicrobial activity against *L. monocytogenes* wild-type strain EGD-e (ATCC BAA-679). At a concentration of 2 × MIC, the growth rate of *L. monocytogenes* was only 44.30% after only 2 h of incubation, and the proportion of late apoptosis cells and necrotic cell was 9.53%, confirming the decrease of cell viability. After MEL-A treatment, the SEM images showed modified irregular cells with rough surfaces and the presence of holes. Moreover, transcriptomic studies revealed that MEL-A determined the upregulation of membrane-associated genes and the downregulation of genes involved in the localization, transport and establishment of localization with an impact on carbohydrate assimilation and the biosynthesis of amino acids.

Another strain of *P. aphidis*, ATCC 32657, was used to obtain a mixture of MEL forms (A, B, C and D), which was tested against biofilms (pre-formed and on co-incubation) of *S. aureus* ATCC 6538 on silicone discs. The biosurfactant reduced biofilm biomass formation within 48 h, decreased cell metabolic activity and had bacteriostatic/bactericidal effect on a 24 h mature biofilm [186].

A MEL-B biosurfactant obtained from *P. tsukubaensis* NBRC1940 acted as a disinfectant agent, inducing inhibition of cell growth of dairy cattle mastitis agents *S. aureus*, with its activity being correlated with the transportation of fatty acids (caprylic acid and myristoleic acid) with antimicrobial activity to the surface of bacterial cells [187]. On the contrary, the biosurfactant had no effect against Gram-negative bacteria *E. coli*, although it bounds on the surface of the cell. This suggests that the lipopolysaccharides from the outer membrane of Gram-negative bacteria might interfere with MEL-B’s capacity to diffuse into the cell.

Also, the efficiency of the antimicrobial activity of MELs seems to be correlated to the alkyl chain length, with the chains containing 8–10 carbons showing a higher antibacterial activity compared to chains of 6, 12 and 14 carbons [188].

Lately, the MELs have also been used for obtaining ecofriendly nanocomposite particles, such as MEL@ZnONP, MEL@AgNP or Ag-ZnO/MEL/GA, exhibiting high activity against food-borne pathogens such as *E. coli*, *S. aureus*, *P. aeruginosa*, *Salmonella* and *C. perfringens* [188].

## 5. Conclusions

In the context in which, during the last years, there was an abusive use of antibiotics or antifungals obtained through chemical synthesis in order to treat microbial infections, their effectiveness was significantly reduced. Also, in the agricultural field/food industry, the conventional methods of controlling contamination with pathogenic/phytopathogenic microorganisms are irrationally used to increase the production of raw material or to extend the shelf life of finished food products to meet the mankind demands. Taking into account all the previously described mechanisms regarding the antimicrobial action of yeasts, it can be considered that they can represent a starting point towards obtaining effective, ecological and innovative strategies to prevent contamination with pathogens or to remove them. Future endeavors for enhancing the described antimicrobial activities of the yeasts would involve the development of new methodologies and improvement of the existing strategies regarding the inoculation/spreading of yeast cultures or proteins with antimicrobial activity, respectively, the use of combinations between antagonistic yeast cultures or proteins and new, emerging synthetic chemicals with proven antifungal or antibacterial action. At last, but not at least, a deepen knowledge of the specific mechanisms of antimicrobial activity correlated with various environmental factors (from natural habitats or industrial processes). As well as the elucidation of involved gene regulation networks, would represent a basis for further practical approaches for using antagonistic yeasts both in biocontrol and biomedicine.

## Figures and Tables

**Table 1:** The most frequently encountered killer toxins secreted by *W. anomalus*.

Killer Toxin	Physico-Chemical Characteristics	Mechanism	Susceptible Strains
PaEXG1 (WaEXG1)	~45.7 kDa; acidic protein and a glycosylation site	Exo-β-1-3 glucanase secreted into the culture medium	Fungal strains that affect fruit quality, e.g., *B. cinerea* [44,45,46]
PaEXG2 (WaEXG2)		Exo-β-1-3 glucanase attached to the cell wall	
WA18	~31 kDa; the producing strain is isolated from dairy products; resistance to high concentrations of sugars, ethanol, pH 2.0–4.5; T > 35 °C	β 1-3 glucanase activity It recognizes β 1-3 and β 1-6 glucan from the cell wal	*B. bruxellensis* [47]
KTCf20	Isolated from wine products; resistance to high concentrations of sugars, ethanol, low pH	β 1-3 glucanase activity It recognizes β 1-3 and β 1-6 glucan from the cell wal	Filamentous fungi affecting wine quality [48,49]
WaF1.712	~140 kDa; the producing strain is isolated from the microbiota of the *Anopheles stephensi* mosquito	β-glucosidase activity	Antiplasmodial action against *Plasmodium berghei* (the etiological agent of malaria) [50]

Apart from *W. anomalus*, other yeasts, including *S. cerevisiae*, have been proven to secrete killer toxins encoded by chromosomal genes.

**Table 2:** Killer yeasts and there antimicrobial activity.

Killer Yeast Species	Killer Toxin	Antimicrobial Properties
*H. uvarum*	K1 KT470	Increases the membrane permeability, forms ion channels
*Z. bailii*	Zygocin KT412	Increases the membrane permeability, forms ion channels
*U. maydis*	KP4 KP6	Increases the membrane permeability, forms ion channels Blocks uptake of Ca^2+^ from the extracellular environment; decreases the intracellular K^+^ concentration
*K. lactis*	Zymocine	When tested against *S. cerevisiae* it degrades ARNt and stops the elongation step of translation represses the *CDC35* gene transcript, stopping the cellular cycle in G1 phase When tested against *Candida*, the mechanisms involves the chitinase activity as well.
*K. wickerhamii*	Kwkt	Unknown
*K. siamensis*	Unknown	Unknown
*W. robertsiae*	pWR1A pWR1B	Most likely cellular arrest in G1 phase
*S.cerevisiae*	K1 K2 K28 KHR KHS CF8 M12	Increases the membrane permeability to H^+^ while releasing K^+^ Activates TOK1 pumps
		Determines cellular arrest in G1 phase
		Not fully established Increases the membrane permeability, forming ion channels
*P. membraniefaciens*	PMKT PMKT2	Activates the HOG signaling path Overexpresses 19 genes involved in maintaining ion homeostasis Increases the membrane permeability for H^+^, K^+^ and Na^+^; induces cellular arrest in the G1 phase of the cellular cycle

**Table 3:** The spectrum of antimicrobial activity of carotenoids produced by red yeasts.

Yeast Strain	Type of Tested Extract	Method Used for Extraction	Potential Sensitive Microbial Strains	Efficiency (MIC Value/Inhibition Zone Diameter)	References
*R. mucilaginosa* 9C3R	Crude extract	Yeast macerate obtained using methanol extraction from PDA cultures was dried and then the crude extract was prepared in dimethyl sulfoxide at the appropriate concentration	*E. coli* ATCC 25922	MIC > 1 mg/mL	[110]
			*S. aureus* ATCC 12600		
			*B. cereus* ATCC 19115		
			*S. thyphimurium* ATCC 14028		
*R. mucilaginosa* YP187	Crude extract	Acetone extraction from freeze-dried cells hydrolyzed with 1N hydrocloric acid	*Enterococcus* sp.	1.9 cm	[60]
			*S. aureus*	2.6 cm	
			*S. faecalis*	2.2 cm	
			*B. subtilis*	2.3 cm	
			*E. coli*	2.9 cm	
			*P. aeruginosa*	2.1 cm	
*R. glutinis*	Crude extract	Pigment extraction using nonpolar solvents such as petroleum ether, n-hexane, ethanol, acetone (25:25:50 *v/v/v*); the colored solution was concentrated and turned into powder using a freeze dryer before determining the antimicrobial activity	*S. aureus* ATCC25923	3.8 µL/mL	[111]
			*S. typhimurium* ST38	15.4 µL/mL	
			*S. aureus* M1	3.8 µL/mL	
			*S. aureus* M2	7.7 µL/mL	
			*S. aureus* M3	3.8 µL/mL	
			*S. aureus* M4	1.9 µL/mL	
			*S. aureus* M5	3.8 µL/mL	
			*S. typhimurium* Ch1	15.5 µL/mL	
			*S. typhimurium* Ch2	15.4 µL/mL	
			*S. typhimurium* Ch3	30.7 µL/mL	
			*S. typhimurium* Ch4	15.4 µL/mL	
			*S. typhimurium* Ch5	15.4 µL/mL	
*R. glutinis* PTCC 5256; *R. glutinis* PTCC5257	Crude extract	Acetone extraction from fresh cellular sediment of wild type strain respectively, mutagenized strain using UV irradiation or due to sodium azide exposure	*S. aureus* PTCC1431	16 mg/mL	[112,113]
			*B. cereus* PTCC1539	8–16 mg/mL	
			*Streptococcus pyogenes* PTCC 1147	16 mg/mL	
			*E. coli* PTCC 1269	32–64 mg/mL	
			*S. enteriditis* PTCC 1709	32–64 mg/mL	
			*Listeria monocytogenes* PTCC1163	16–64 mg/mL	
			*Alternaria citri* ATCC	128 mg/mL	
			*P. digitatum* ATCC 48.113	64–128 mg/mL	
*R. mucilaginosa* CCMA 0156	Crude extract	Acetone: methanol extraction from dried cell mass	*P. aeruginosa*	1.225–1.6 mg/mL	[114]
			*S. cholerasus*	0.612–1.225 mg/mL	
			*E. coli*	1.225–1.6 mg/mL	
			*S. aureus*	0.612–1.225 mg/mL	
			*L. monocytogenes*	1.225–1.6 mg/mL	
*R. diobovata* R1	β-carotene (0.03 mg/mL)	Acetone extraction in presence of magnetic beads followed by chromatographic purification. The antimicrobial activity was determined for 0.03 mg/mL pure β-carotene	*C. albicans* HAM25	2.8 cm	[115]
			*C. dubliniensis*	2.9 cm	
			*C. tropicalis* HAM13	2.7 cm	
			*Cutaneotrichosporon dermatis* Judy 4	2.3 cm	
*R. mucilaginosa* R2	β-carotene (0.03mg/mL)		*C. albicans* HAM25	3.0 cm	
			*C. dubliniensis*	2.7 cm	
			*C. tropicalis* HAM13	2.9 cm	
			*C. dermatis* Judy 4	2.4 cm	
*R. rubra*	Torularhodin	Alkaline methanol extraction from cells with permeabilized cell wall	*S. aureus* ATCC25923	22.18 µg/L	[116,117]
			*E. faecalis* ATCC29212	44.375 µg/L	
			*E. coli* K 120MG1655	22.18 µg/L	
			*C. utilis*	44.375 µg/L	
			*A. ochraceus*	44.375 µg/L	
			*F. oxysporum* MUCL 791	44.375 µg/L	

**Table 4:** The biocontrol activity of yeast produced glycolipids.

Glycolipid Biosurfactants	Producing Species (Strain)	Biocontrol Activity	Fungal Species Affected
Sophorolipids (SL)	*Starmerella bombicola* ATCC 22214	inhibition of fungal mycelial growth	*Aspergillus flavus* *Aspergillus melleus* *Aspergillus ochraceus* *Aspergillus parasiticus* *Aspergillus niger* *Fusarium oxysporum* *Botrytis cinerea* *Rhizopus* spp. *Pythium ultimum* *Sclerotium rolfsii* *Rhizoctonia solani*
		modifications of mycelia morfology	*Fusarium oxysporum* *Aspergillus niger* *Botrytis cinerea* *Rhizopus* spp.
		modification of cell and hyphae appearance	*Botrytis cinerea* *Rhizoctonia solani* *Pythium ultimum* *Sclerotium rolfsii*
	*Wickerhamiella domercqiae* Y_2A_	inhibition of fungal mycelial growth and spore germination modification of hyphae appearance	*Fusarium* sp. *Fusarium oxysporum* *Fusarium concentricum* *Pythium ultimum* *Pyricularia oryzae* *Rhizoctorzia solani* *Alternaria kikuchiana Gaeumannomyces graminis* var. *tritici* *Phytophthora infestans*
	*Rhodotorula babjevae* YS3	inhibition of fungal growth	*Colletotrichum gloeosporioides*
	*Rhodotorula glutinis*	inhibition of fungal growth	*Aspergillus niger* *Alternaria* sp. *Rhizopus* sp. *Syncephalastrum* sp.
Mannosylerytrithol lipids (MEL)			
MEL-A	*Pseudozyma* sp.	supression of conidia germination	*Blumeria graminis* f. sp. *tritici*
MEL-B	*Pseudozyma* sp.	supression of conidia germination	*Colletotrichum dematium* *Magnaporthe grisea*
MEL-C	*Pseudozyma* sp.	supression of conidia germination	*Colletotrichum dematium*
